# Improved Small Molecule Identification through Learning Combinations of Kernel Regression Models

**DOI:** 10.3390/metabo9080160

**Published:** 2019-08-01

**Authors:** Céline Brouard, Antoine Bassé, Florence d’Alché-Buc, Juho Rousu

**Affiliations:** 1 Unité de Mathématiques et Informatique Appliquées de Toulouse, UR 875, INRA, 31326 Castanet Tolosan, France; 2 LTCI, Télécom Paris, Institut Polytechnique de Paris, 75634 Paris, France; 3 Helsinki Institute for Information Technology HIIT, Department of Computer Science, Aalto University, 00076 Espoo, Finland

**Keywords:** metabolite identification, machine learning, structured prediction, kernel methods

## Abstract

In small molecule identification from tandem mass (MS/MS) spectra, input–output kernel regression (IOKR) currently provides the state-of-the-art combination of fast training and prediction and high identification rates. The IOKR approach can be simply understood as predicting a fingerprint vector from the MS/MS spectrum of the unknown molecule, and solving a pre-image problem to find the molecule with the most similar fingerprint. In this paper, we bring forward the following improvements to the IOKR framework: firstly, we formulate the IOKRreverse model that can be understood as mapping molecular structures into the MS/MS feature space and solving a pre-image problem to find the molecule whose predicted spectrum is the closest to the input MS/MS spectrum. Secondly, we introduce an approach to combine several IOKR and IOKRreverse models computed from different input and output kernels, called IOKRfusion. The method is based on minimizing structured Hinge loss of the combined model using a mini-batch stochastic subgradient optimization. Our experiments show a consistent improvement of top-k accuracy both in positive and negative ionization mode data.

## 1. Introduction

In recent years, the massively increased amounts of publicly available reference tandem mass (MS/MS) spectra in databases such as GNPS [1] and MassBank [2] have caused a revolution in small molecule identification. In particular, the use of modern machine learning approaches has become feasible [3], and led to the generation of a host of machine learning approaches and identification tools such as FingerID [4,5], CFM-ID [6,7], CSI:FingerID [8], CSI:IOKR [9], magnitude-preserving IOKR [10] ChemDistiller [11], SIMPLE [12], ADAPTIVE [13] and SIRIUS [14]. The identification rates have witnessed a step-change upward, and consequently the use of the tools in practical work-flows has massively increased (see, e.g., [15]).

The majority of the machine learning methods rely on the same conceptual scheme [3] introduced with FingerID [4]: predicting molecular fingerprints from MS/MS data and finding the most similar fingerprint from the molecular structure database. This approach has been very successful, for example, CSI:FingerID [8] and CSI:IOKR [9] have been top performers in the most recent CASMI contests (2016: [16] and 2017: [17]). The alternative conceptual approach for small molecule identification, sometimes called in silico fragmentation [3], calls for predicting MS/MS spectra for a set of candidate molecular structures and choosing the most similar predicted MS/MS spectrum to the observed MS/MS spectrum. This approach is used, e.g., in the non-machine learning based MetFrag [18,19] as well as CFM-ID [6,7], which is the most notable machine learning tool relying on the in silico fragmentation approach.

CSI:FingerID uses an array of Support Vector Machines with multiple kernel learning [20] to individually predict each bit of the molecular fingerprint vector, whereas CSI:IOKR predicts the molecular structures through a single structured output prediction [21] algorithm, called Input–Output Kernel Regression (IOKR) [22], where both inputs and outputs are kernelized for the best performance. Due to this approach, CSI:IOKR is extremely fast to train and is on par with CSI:FingerID in accuracy. Both CSI:FingerID and CSI:IOKR make use of multiple data sources, fused using the multiple kernel learning (MKL) algorithm ALIGNF [23] that sets importance weights to the input kernels prior learning the fingerprint prediction models. Interestingly, CSI:FingerID [8] benefits from the MKL technique more than CSI:IOKR [9] that provides equally good or better results using uniform weights for the inputs, a technique referred to as *uniform* MKL or Unimkl. This corresponds to summing up or averaging the input kernels.

In this paper, we bring forward two methodological contributions. Firstly, we extend the IOKR [9] approach by formulating essentially an in silico fragmentation problem which we call IOKRreverse. From a set of candidate molecular structures, we implicitly (through a kernel function) predict a representation of an MS/MS spectrum for each candidate, and solve a pre-image problem to output the molecular structure whose predicted MS/MS is the closest to the observed one. All this computation is done through kernel matrices of the inputs (MS/MS spectra) and outputs (molecular structures).

Secondly, we introduce an approach called IOKRfusion to combine multiple IOKR and IOKRreverse models, which arise from the use of different input and output kernels on the training data. The models are combined by minimizing the structured Hinge loss [24], which is frequently used in structured output learning, and corresponds to a convex (thus efficiently computable) upper bound for maximizing top-1 accuracy over a candidate set (an NP-hard task). We bring forward a mini-batch subgradient algorithm for the optimization. This way of aggregating multiple data sources is sometimes called *late fusion*, since the model learning happens before the aggregation, as compared to the multiple kernel learning using ALIGNF [23], which happens before model learning, making it an *early fusion* approach.

The structure of the paper is as follows. In Section 2, we review the IOKR model and present the IOKRreverse model as well as the late fusion approach for minimizing the structured Hinge loss of the combined model. Section 3 presents our experiments with the models and Section 4 presents the discussion.

## 2. Materials and Methods

In the following, we note X the set of tandem mass spectra and Y a set containing 2D molecular structures. We consider a set of *ℓ* training examples ${\left\{x_{i},y_{i}\right\}}_{i=1}^{\ell }\subseteq X\times Y$.

### 2.1. Input–Output Kernel Regression

Input Output Kernel Regression (IOKR) [22] is a machine learning framework that can be used for solving structured prediction problems. Structured output prediction involves the prediction of outputs corresponding to complex structured objects, for example graphs or trees, rather than scalar values as in regression and classification problems. Structure output prediction can also be used in the case where we search to predict multiple interdependent outputs. Structured data can generally be decomposed into several parts and structured prediction approaches make use of the dependencies existing between these parts.

The IOKR framework has been used in CSI:IOKR [9] for compound structure identification (CSI), where we search to predict the molecular structures of metabolites from their MS/MS spectra. In [9], the similarities between the molecular structures were encoded using an output kernel function $k_{y}:Y\times Y\to R$. This kernel is associated with a high-dimensional vector space $F_{y}$, referred to as the output feature space, and a function $\psi :Y\to F_{y}$ that maps outputs (molecules) to the output feature space $F_{y}$. The inner product in the feature space can be evaluated by computing the values of the output kernel:

$${\langle \psi \left(y\right),\psi \left(y^{\prime }\right)\rangle }_{F_{y}}=k_{y}\left(y,y^{\prime }\right),\;\;\;\forall y,y^{\prime }\in Y.$$

IOKR solves the metabolites identification problem by first learning a function *h* from X to $F_{y}$ that approximates the output feature map ψ. *h* is learned by solving the following regression problem:

(1) $$\min_{h\in H}\sum_{i=1}^{\ell }∥h\left(x_{i}\right)-\psi \left(y_{i}\right){∥}_{F_{y}}^{2}+\lambda_{h}{∥h∥}_{H}^{2},$$

where $\lambda_{h}>0$ is a regularization parameter. *h* is modeled as: h(x)=Wϕ(x),∀x∈X where $\phi :X\to F_{x}$ is a feature map from X to an input feature space $F_{x}$ associated with an input kernel k:X×X→R measuring a similarity between MS/MS spectra. *W* is a linear operator from $F_{x}$ to $F_{y}$. When using this model, the solution to Problem (1) is given by:

(2) $$h\left(x\right)=\sum_{i=1}^{\ell }\alpha_{i}\left(x\right)\psi \left(y_{i}\right),\;\;\mathrm{with}\alpha \left(x\right)={\left(\lambda_{h}I_{\ell }+K_{X}\right)}^{-1}k_{X}^{x},$$

where $K_{X}$ is the kernel matrix of $k_{x}$ on the training set: ${\left[K_{X}\right]}_{i,j}=k_{x}\left(x_{i},x_{j}\right)\;\forall i,j=1,\ldots ,\ell$ and $k_{X}^{x}={\begin{array}{c} \left[\begin{array}{c} k_{x}\left(x_{1},x\right),\cdots k_{x}\left(x_{\ell },x\right) \end{array}\right] \end{array}}^{T}\in R^{\ell }$ collects the kernel evaluations of the training inputs against *x*.

Given the prediction $h(x_{i})$ for an MS/MS spectrum $x_{i}$, the prediction of the corresponding molecule $y_{i}$ requires solving a pre-image problem. For this, we consider a subset $Y_{x_{i}}$ of molecular structures from a large database such as PubChem [25], for example the set of molecules having the same molecular formula as $x_{i}$ if it is known or having a similar mass to the one measured for $x_{i}$. We then search for the nearest molecule to the prediction $h(x_{i})$ in the output feature space $F_{y}$:

$$f\left(x_{i}\right)=\underset{y\in Y_{x_{i}}}{\mathrm{argmin}}{∥h\left(x_{i}\right)-\psi \left(y\right)∥}_{F_{y}}^{2}.$$

When replacing $h(x_{i})$ by the solution given in (2), this can be rewritten as follows:

$$f\left(x_{i}\right)=\underset{y\in Y_{x_{i}}}{\mathrm{argmin}}\alpha {\left(x_{i}\right)}^{T}K_{Y}\alpha \left(x_{i}\right)+k_{y}\left(y,y\right)-2\alpha {\left(x_{i}\right)}^{T}k_{Y}^{y},$$

where $K_{Y}$ and $k_{Y}^{y}$ are defined similarly to $K_{X}$ and $k_{X}^{x}$.

### 2.2. IOKRreverse: Mapping Kernel Representations of Molecules to Kernel Representation of MS/MS Spectra

In this subsection, we introduce a variant of IOKR called IOKRreverse inspired from recent works designed to remedy the problem of hubness in high dimensional nearest-neighbour approaches [26]. Hubness in k-nearest neighbours refers to the emergence of hubs, i.e., points that are among the k-nearest neighbours of a lot of data. The presence of hubs can have a bad impact on k-nearest neighbours accuracy. Recently, this phenomenon observed in high dimensional search spaces has also been identified to be an important source of error in Zero-Shot Learning [27]. In a nutshell, Zero-Shot Learning [28,29] is a realistic machine learning setting for multi-class classification especially meaningful when the number of classes is extremely large: it consists of learning a classifier able to predict classes not seen during the training phase. One of the most relevant approaches to zero-shot learning relies on a regression-based scheme very similar to IOKR. Labels are first mapped onto a Euclidean space. Then, a function is learned to solve the regression problem in the Euclidean space instead of solving a classification problem. Eventually, to make a prediction on a new example, a nearest neighbour search provides the label closest to the mapped example. Recent contributions [27] have shown that reversing the regression problem, meaning attempting to approximate the relationship between the outputs (in this case the mapped labels) and the inputs (for instance images) allows for mitigating the hubness problem and provides a significant accuracy improvement. Variance of the data on which the nearest neighbour search is performed is key to the hubness. As regression has a shrinkage effect on mapped data impacting their variance, direct regression and reverse regression do not have the same effect. The authors in [27] have demonstrated that reverse regression is expected to provide smaller variance and better performance when retrieving the output objects.

**Figure 1:**
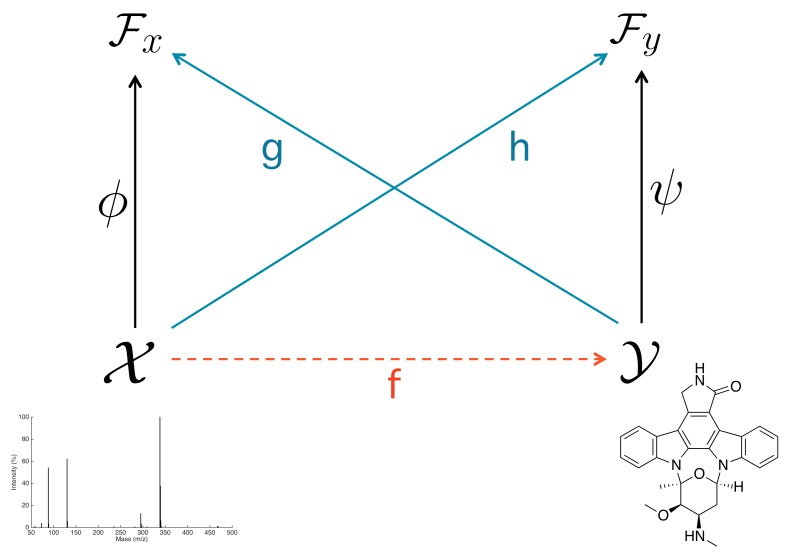
Schematic illustration of IOKR and IOKRreverse approaches. IOKR learns a function *h* to map MS/MS spectra to a molecular feature space $F_{y}$, whereas IOKRreverse learns a function *g* to map the molecular structures to a MS/MS feature space $F_{x}$.

As the pre-image problem in IOKR boils down to search for the nearest neighbour, we propose to adopt a similar scheme in the context of IOKR. Instead of learning a function *h* that maps the input examples to the output feature space, we learn a function *g* that maps the output examples to the input feature space, in this case, molecular structures to MS/MS feature space (see Figure 1).

To learn this function *g*, we solve the following optimization problem:

(3) $$\min_{g\in G}\sum_{i=1}^{\ell }∥g\left(y_{i}\right)-\phi \left(x_{i}\right){∥}_{F_{x}}^{2}+\lambda_{g}{∥g∥}_{G}^{2},\;\lambda_{g}>0.$$

This time the function *g* is modeled as g(y)=Vψ(y),∀y∈Y, where *V* is a linear operator from $F_{y}$ to $F_{x}$. When replacing g(y) by this expression, the solution of the IOKRreverse optimization problem in (3) is given by:

$$g\left(y\right)=\sum_{i=1}^{\ell }\beta_{i}\left(y\right)\phi \left(x_{i}\right),\;\;\mathrm{where}\beta \left(y\right)={\left(\lambda_{g}I_{\ell }+K_{Y}\right)}^{-1}k_{Y}^{y}.$$

In IOKRreverse, the pre-image problem consists of solving a nearest neighbor problem in the input feature space $F_{x}$. Given the input feature vector of an MS/MS spectrum $x_{i}$, we use the function *g* learned in the previous step for predicting the input feature vectors for all the candidates in $Y_{x_{i}}$ (the candidate set of $x_{i}$). We then search the closest candidate to $\phi (x_{i})$ in the input feature space $F_{x}$:

$$f\left(x_{i}\right)=\underset{y\in Y_{x_{i}}}{\mathrm{argmin}}{∥g\left(y\right)-\phi \left(x_{i}\right)∥}_{F_{x}}^{2}.$$

Using the kernel trick in the input space, this can be rewritten under the following form:

$$f\left(x_{i}\right)=\underset{y\in Y_{x_{i}}}{\mathrm{argmin}}\beta {\left(y\right)}^{T}K_{X}\beta \left(y\right)+k_{x}\left(x_{i},x_{i}\right)-2\beta {\left(y\right)}^{T}k_{X}^{x_{i}}.$$

### 2.3. Combining Multiple Models to Maximize Top-1 Accuracy

Combining multiple representations and data sources is a potent way of improving the predictive capabilities of machine learning models. This task can be implemented in several ways [30], in particular using early fusion, where data sources and representations are combined prior to learning the model, or late fusion, where the models learned using different representations are combined after model learning. Given multiple input kernels, a popular early fusion approach is to use multiple kernel learning [23] to find a liner combination of the input kernels so that the combined kernel would be similar to a given target kernel. The learned combined kernel is then used as the input kernel in the next phase. This approach has been previously used in both CSI:FingerID [14] and CSI:IOKR [9].

Here, we propose instead to combine the set of models learned by using individual input and output kernels after learning the models, that is, using late fusion. Several models are learned using the IOKR and IOKRreverse approaches for different pairs of input, output kernels. For each of these models, we can compute a compatibility score s(x,y) for an input–output pair (x,y)∈X×Y, for example using the normalized cosine similarity:

$$\begin{array}{cc} s(x,y) & =\frac{{\langle h(x),\psi (y)\rangle }_{F_{y}}}{∥h(x)∥∥\psi (y)∥}\mathrm{in}\mathrm{the}\mathrm{case}\mathrm{of}\mathrm{IOKR},\\ s(x,y) & =\frac{{\langle g(y),\phi (x)\rangle }_{F_{x}}}{∥g(y)∥∥\phi (x)∥}\mathrm{for}\mathrm{IOKRreverse}. \end{array}$$

We then search to learn a linear combination of the score functions obtained with IOKR and IOKRreverse: $\sum_{k=1}^{K}w_{k}s_{k}\left(x,y\right)$. The goal is to learn a vector w such that the linear combination of the scores for the correct pair $\left(x_{i},y_{i}\right)$ is separated from the combined scores of all incorrect pairs $\left(x_{i},y\right)$ for $y\in Y_{x_{i}}$. For this, we solve the optimization problem proposed in the structured SVM approach [31]:

(4) $$\min_{w}J\left(w\right)=\frac{\lambda }{2}{∥w∥}^{2}+\frac{1}{\ell }\sum_{i=1}^{\ell }\ell \left(w,x_{i},y_{i}\right),\;\lambda \geq 0,$$

where

$$\ell \left(w,x_{i},y_{i}\right)=\max_{y\in Y_{x_{i}}}\left(\Delta \left(y_{i},y\right)-w^{T}(s\left(x_{i},y_{i}\right)-s\left(x_{i},y\right)\right)$$

is the structured Hinge loss and $s\left(x,y\right)={\left(s_{1}\left(x,y\right),\ldots ,s_{K}\left(x,y\right)\right)}^{T}$ is a vector of compatibility scores. $\Delta (y_{i},y)$ is a measure of distance between the two output structures $y_{i}$ and *y*. Here, we used the Hamming loss between molecular fingerprints: $\Delta \left(y_{i},y\right)=\frac{1}{d}\sum_{j=1}^{d}1_{fp\left(y_{i}\right)\neq fp\left(y\right)}$. We solve this optimization problem using a mini-batch subgradient descent (see Algorithm 1). This is an iterative optimization algorithm. At each step, a mini batch B of *m* training examples is selected at random and the weights are updated as follows:

$$w^{\left(k+1\right)}=w^{\left(k\right)}-t\frac{1}{\left|B\right|}\sum_{i\in B}{▽}_{w}J_{i}\left(w\right),$$

where *t* is the step size and $J_{i}\left(w\right)=\frac{\ell \lambda }{2}{∥w∥}^{2}+\ell \left(w,x_{i},y_{i}\right)$.

**Algorithm 1:**Mini-batch subgradient descent for the score aggregation.

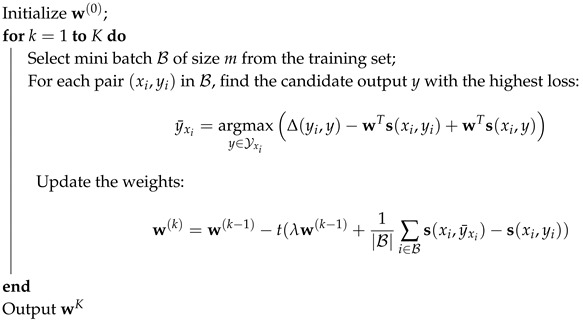



### 2.4. Kernels

In this subsection, we present the different input and output kernels used to measure the similarity between MS/MS spectra and molecular structures, respectively.

#### 2.4.1. Input Kernels

We considered 15 different kernels on MS/MS spectra (listed in Table 1). Among these kernels, 14 were defined based on fragmentation trees [32,33] that model the fragmentation process of the metabolites as a tree. In this tree, each node corresponds to a peak in the MS/MS spectrum and is annotated by the predicted molecular formula for the corresponding fragment. The edges correspond to losses and give information about the fragmentation reactions existing between pairs of fragments. Different kernels on fragmentation trees can be defined by comparing the nodes, the edges or the paths for pairs of fragmentation trees. In addition, we used the recalibrated probability product kernel (PPKr) [4,8] that models the peaks in an MS/MS spectrum as a two-dimensional, normal distribution with $\sigma_{m}$ and $\sigma_{i}$ the standard deviations on the mass-to-charge ratio and the intensity. This kernel writes as:

$$k_{x}^{PPKr}\left(x,x^{\prime }\right)=\frac{1}{n_{x},n_{x^{\prime }}}\frac{1}{4\pi \sigma_{m}\sigma_{i}}\sum_{\ell ,\ell^{\prime }=1}^{n_{x},n_{x^{\prime }}}\exp\left(-\frac{{\left(m\left(x_{\ell }\right)-m\left(x_{\ell^{\prime }}^{\prime }\right)\right)}^{2}}{4\sigma_{m}^{2}}\right)\exp\left(-\frac{{\left(i\left(x_{\ell }\right)-i\left(x_{\ell^{\prime }}^{\prime }\right)\right)}^{2}}{4\sigma_{i}^{2}}\right),$$

where $m(x_{\ell })$ denotes the mass-to-charge ratio of the *ℓ*-th peak of spectrum *x* and $i(x_{\ell })$ its intensity. $n_{x}$ indicates the number of peaks contained in *x*.

#### 2.4.2. Output Kernels

We measured similarities between molecular structures by using kernels between molecular fingerprints. A molecular fingerprint represents the structure of a molecule as a binary vector, where each value indicates the presence or absence of a molecular property. A molecular property can encode the presence of a certain bond, substructure or atom configuration. As in [9], we used a linear and a Gaussian kernel on molecular fingerprints. In addition, we considered the Tanimoto kernel [35] that is commonly used for comparing molecular fingerprints:

$$k_{y}^{tan}\left(y,y^{\prime }\right)=\frac{|fp\left(y\right)\cap fp\left(y^{\prime }\right)|}{|fp\left(y\right)\cup fp\left(y^{\prime }\right)|}=\frac{fp{\left(y\right)}^{T}fp\left(y^{\prime }\right)}{{∥fp\left(y\right)∥}^{2}+{∥fp\left(y^{\prime }\right)∥}^{2}-fp{\left(y\right)}^{T}fp\left(y^{\prime }\right)}.$$

We also proposed a modified version of the Gaussian kernel, in which the distance is replaced by the distance between the feature vectors associated with the Tanimoto kernel:

$$\begin{array}{ll} k_{y}^{gauss-tan}\left(y,y^{\prime }\right) & =\exp\left(-\gamma {∥\psi^{tan}\left(y\right)-\psi^{tan}\left(y^{\prime }\right)∥}_{F_{y}}^{2}\right)\\ & =\exp\left(-\gamma \left(k_{y}^{tan}\left(y,y\right)+k_{y}^{tan}\left(y^{\prime },y^{\prime }\right)-2k_{y}^{tan}\left(y,y^{\prime }\right)\right)\right). \end{array}$$

## 3. Results

We used two subsets of tandem mass spectra from GNPS (Global Natural Products Social molecular networking) [1] and MassBank [2] to evaluate the performance of our method. The first subset contains 6974 MS/MS spectra, corresponding to 6504 structures, measured with a positive ionization mode while the second subset contains 3578 MS/MS spectra, corresponding to 2376 structures, measured with a negative ionization mode. In the positive ionization mode, the spectra have the following adducts: [M + H]${}^{+}$, [M + K]${}^{+}$, [M + Na]${}^{+}$, [M − H2O + H]${}^{+}$, [M]${}^{+}$ and [M + H3N + H]${}^{+}$, while the following adducts are observed in the negative ionization mode: [M − H]${}^{-}$, [M]${}^{-}$, [M + Cl]${}^{-}$, [M − H2O − H]${}^{-}$, [M + CH2O2 − H]${}^{-}$ and [M + C2H4O2 − H]${}^{-}$. We consider separately the MS/MS spectra measured with negative and positive ionization modes as the mechanisms of fragmentation of positive and negative ions are different. We visualize this difference in the Supplementary Materials by comparing the spectra similarities in the different ionization modes.

The spectra correspond to LC-MS/MS data measured with Quadrupole-Time of Flight (Q-ToF), Orbitrap, Fourier Transform Ion Cyclotron Resonance (FTICR) and ion trap instruments. The MS/MS spectra measured with different collision energies have been merged together. We considered molecular fingerprints containing 7593 molecular properties computed using the Chemistry Development Kit (CDK) [36]. These fingerprints contain molecular properties from FP2 (55 bits), FP3 (307 bits), MACCS (166 bits), Pubchem fingerprint (881 bits), Klekota–Roth (4860 bits) [37] and ECFP (Extended-connectivity Fingerprints) (1324 bits) [38].

### 3.1. Experimental Protocol

The performance of the models were evaluated using 5-fold cross-validation (CV). The MS/MS spectra corresponding to the same molecular structures were contained in the same fold. This avoids having the case where an MS/MS spectrum in the training set has the same molecular structure as a test example. In each round of the cross-validation, we used three folds for training the IOKR and IOKRreverse models, one fold to train the score aggregator and the last fold as test set. We evaluated the performance by computing the averaged top-*k* accuracy over the test examples. This corresponds to the percentage of test examples for which the true molecular structure was found among the *k* top ranked molecules.

The regularization parameters $\lambda_{h}$ and $\lambda_{g}$ were tuned on the training set of each fold among a grid. We selected the parameters that minimize the averaged mean squared error. Regarding the parameter λ used in the aggregation model, it was selected on the validation set using 4-CV such that it maximized the top-1 accuracy. Regarding the parameter γ of the Gaussian and Gaussian–Tanimoto output kernels, we took the value for which the entropy is maximal. All of the kernels have been centered and normalized.

In the pre-image, we assumed the molecular formula of the test spectra to be known and we considered the molecules from Pubchem with the same molecular formula as candidates.

For the Mini-batch subgradient descent, we used 30 epochs, this means that we passed on the full training set 30 times. In each epoch, the training set was split randomly into small batches of fixed size. The mini-batch size was selected by cross-validation on the validation set. The learning rate was set to $t=\frac{1}{\lambda k}$ at iteration *k*.

In Section 3.4, we include the predictive performance obtained with the competing method CSI:FingerID [8]. These results were obtained using the CSI:FingerID 1.1 version with the modified Platt scoring, for which the best predictive performance have been observed in [8]. We applied this method on the same cross-validation folds, using four folds for training and the last fold for testing, and the parameter *c* was tuned on the training sets. We used as input kernel the combination of the 15 input kernels learned with the ALIGNF algorithm [23].

### 3.2. Results Obtained with IOKR and IOKRreverse Using Different Kernels

**Figure 2:**
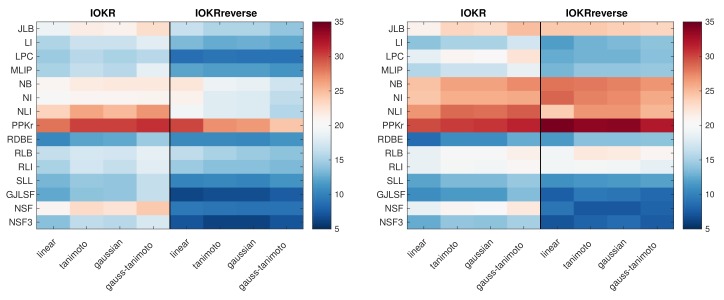
Heatmap of the top-1 accuracy obtained with IOKR and IOKRreverse for different input and output kernels in the negative ionization mode (**a**) and the positive ionization mode (**b**). The rows correspond to the different input kernels while the columns indicate the output kernels.

We first report on using IOKR and IOKRreverse as standalone models, and selecting a single input and output kernel at the time for the model. In Figure 2, we visualized the top-1 accuracy obtained with the IOKR and IOKRreverse approaches using different pairs of single input and single output kernel. The best input kernel is PPKr, consistently for both IOKR and IOKRreverse, for both ionization modes and different output kernels. In the case of negative ionization mode, the predictive performance obtained with IOKRreverse is worse than the ones obtained with IOKR for most of the kernels. With the PPKr kernel, the top-1 accuracy of IOKRreverse using a linear output kernel is equal to 29.85, slightly below the highest top-1 accuracy obtained with IOKR (30.74). In addition, the fragmentation tree based kernels do not work well as standalone input kernels in negative ionization mode, in particular for IOKRreverse. In the positive ionization mode, the results are different. IOKR still performs better for most of the kernels. However, IOKRreverse is better than IOKR for three out of the four best input kernels (NB, NI, NLI, PPKr). This improvement is especially important for the best performing input kernel PPKr. When using this input kernel, IOKRreverse increases the top-1 accuracy by three percentage points: 34.36 instead of 31.22 for IOKR.

### 3.3. Weights Learned by the Aggregation Model

**Figure 3:**
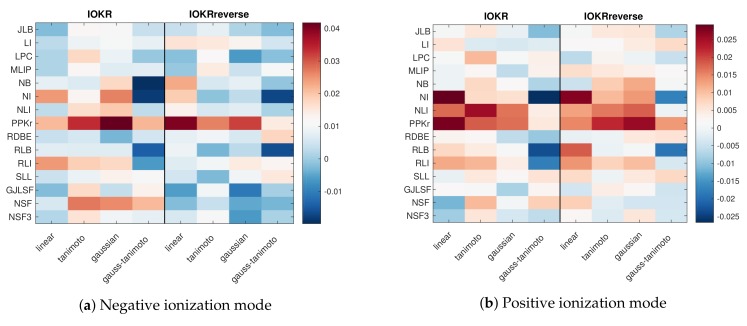
Heatmap of the weights learned during the score aggregation in the negative ionization mode (**a**) and the positive ionization mode (**b**). The weights have been averaged over the five cross-validation folds.

Next, we turn our attention to the proposed score aggregation method (IOKRfusion). We first visualize the weights learned for the individual models in the two combined models (we have separate negative and positive mode models). The weights are shown in Figure 3.

We first notice that all models with PPKr as the input kernel are highly weighted in the combined model, regardless of the output kernel or whether IOKR or IOKRreverse is used. This is true for both combined models (positive and negative modes). In addition, the high aggregation weights tend to appear for models that are good predictors in the standalone setting as well (c.f. Figure 2), indicating that the models have complementarity besides good individual performance.

### 3.4. Results for Combined Models

Next, we report on the predictive performance of different aggregated models, including the early fusion MKL approaches and the proposed IOKRfusion approach relying on late fusion. We compare the results of score aggregation to IOKR Unimkl and CSI:FingerID. IOKR Unimkl denotes using IOKR with a linear combination of the 15 kernels as the single input kernel. In this combination, the same weight, 1, is given to each input kernel. This approach has previously shown to be a competitive way to combine input kernels for IOKR models [9]. For IOKRfusion, we show separately the top-k accuracy of the model restricted to combining the 60 IOKR models (4*15) and the model restricted to combining the 60 IOKRreverse models, as well as the combination including both IOKR and IOKRreverse models. The results obtained are shown in Table 2.

We first note that IOKRfusion aggregating all scores (IOKR and IOKRreverse) gives the best results by a significant margin in both negative and positive mode. Interestingly, the two restricted models, IOKRfusion aggregating either IOKR scores or IOKRreverse scores, do not give a consistent improvement over the IOKR Unimkl variants. Among the IOKR Unimkl variants, there is no clear winner: positive and negative mode seem to favor different output kernels, but the differences are relatively small.

**Figure 4:**
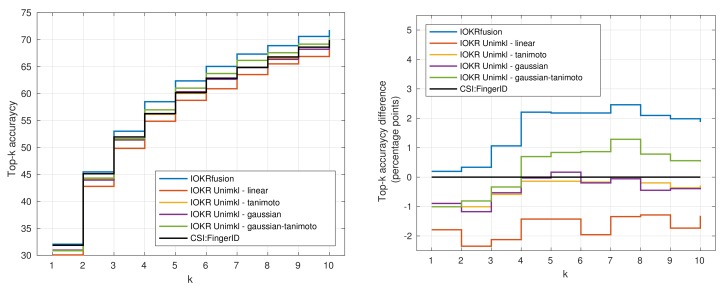
Plot of the top-k accuracy for IOKR Unimkl, CSI:FingerID and IOKRfusion in the negative ionization mode.

**Figure 5:**
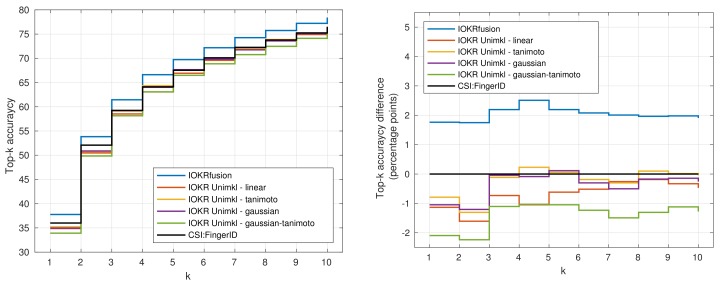
Plot of the top-k accuracy for IOKR Unimkl, CSI:FingerID and IOKRfusion in the positive ionization mode.

In Figure 4 and Figure 5, we visualize the top-k accuracies of IOKR Unimkl and CSI:FingerID with the combination of all scores (IOKRfusion) in the negative (Figure 4) and positive (Figure 5) ionization modes. The plots in these figures also represent the top-k accuracy difference compared to the top-k accuracy obtained with CSI:FingerID. We observe that IOKRfusion consistently obtains better results than the other approaches. In the positive ionization mode, the score aggregation model improves upon CSI:FingerID and all the IOKR Unimkl models by around two percentage points for top-1 to top-10 accuracy. In the negative ionization mode, we observe a similarly consistent increase of one percentage point compared to the other models.

### 3.5. Running Times

We evaluated the running times of the different approaches on the negative dataset using 2859 spectra in the training set and 719 spectra in the test set (see Table 3). In this evaluation, we fixed the values of the different hyperparameters. In the score aggregation algorithm, we set the batch size to 15 and the number of epochs to 30. For the IOKR and IOKRreverse models that use a single kernel in input, we evaluated the running times for each of the 15 input kernels and averaged the training and test times. All of the models were trained on a single computer without any GPU acceleration or special infrastructures.

From the table, we can see that all single kernel IOKR models are trained in less than 10 s each, while computing the predictions (computing the pre-image) takes the majority of the time, 1–10 min depending on the output kernel. IOKRreverse models are equally fast to train, but the predictions are heavier to compute than for IOKR, the time consumed being in the interval of 28–35 min depending on the output kernel. The models based on multiple kernel learning (Unimkl) are only slightly more demanding to train, 4–12 s per model.

Computing the IOKRfusion model is comparatively very efficient after the component models have been trained and their predictions extracted. Learning a combination of 120 models: 15 (input kernels) × 4 (output kernels) × 2 (IOKR and IOKRreverse models), took slightly over three minutes, corresponding to around 1.5 s amortized time per model. Testing is much faster still, taking only 0.1 s in total, starting from the scores of the individual models.

The running times for CSI:FingerID are not included, but it has been shown in [9] that IOKR Unimkl is approximately 7000 times faster to train and is faster to test than CSI:FingerID.

## 4. Discussion

In this paper, we presented extensions to the IOKR framework that were shown to improve the identification rates of molecular structures from the MS/MS data. The first extension, IOKRreverse, changes the learning setting so that the regression problem is performed in the input (MS/MS) feature space rather than the output feature space. Using IOKRreverse as a standalone model in the positive ionization mode improved the IOKR results, but similar behaviour was not observed in the negative ionization mode.

The IOKRreverse model, which with a slight abuse of concepts, could be thought as a ‘in silico fragmentation model’ in that the model implicitly predicts a feature map of an MS/MS spectrum from the molecular structure. However, we must stress that there is no explicit fragmentation model present in IOKRreverse. Implicit fragmentation model could be seen if the input kernel is based on a fragmentation tree. Then, IOKRreverse can be interpreted as mapping molecular structures into a feature space where fragmentation trees are embedded, and the pre-image problem finds the molecular structure whose predicted fragmentation tree embedding is closest to the predicted one.

The proposed IOKRfusion approach, which combines several IOKR and IOKRreverse models trained with individual input and output kernels, obtained the best results in both negative and positive ionization mode, showing the potential of the approach. In particular, we note the late fusion approach, used by IOKRfusion, improves over the early fusion MKL approach Unimkl, which was previously found to be the best choice for CSI:IOKR [9]. In addition, the IOKRfusion approach turned out to outperform CSI:FingerID, which relies on an ALIGNF algorithm for MKL early fusion. The proposed IOKRfusion approach is also extremely fast to train and test. The dominant time cost is the extraction of the predictions of the individual models to be combined. In conclusion, IOKRfusion can be seen to maintain the computational efficiency of the IOKR framework, while improving the small molecule identification accuracy.

## Figures and Tables

**Table 1:** Description of the input kernels (see [34] for further details).

	Name	Description
LI	Loss intensity	counts the number of common losses weighted by the intensity
RLB	Root loss binary	counts the number of common losses from the root to some node
RLI	Root loss intensity	weighted variant of RLB that uses the intensity of terminal nodes
JLB	Joined loss binary	counts the number of common joined losses
LPC	Loss pair counter	counts the number of two consecutive losses within the tree
MLIP	Maximum loss in path	counts the maximum frequencies of each molecular formula in any path
NB	Node binary	counts the number of nodes with the same molecular formula
NI	Node intensity	weighted variant of NB that uses the intensity of nodes
NLI	Node loss interaction	counts common paths and weights them by comparing the molecular formula of their terminal fragments
SLL	Substructure in losses and leafs	counts for different molecular formula in how many paths they are conserved (part of all nodes) or cleaved off intact (part of a loss)
NSF	Node subformula	considers a set of molecular formula M and counts how often each of them occurs as subset of nodes in both trees
NSF3		takes the value of NSF to the power of three
GJLSF	Generalized joined loss subformula	counts how often each molecular formula from M occurs as subset of joined losses in both fragmentation graphs
RDBE	Ring double-bond equivalent	compares the distribution of ring double-bond equivalent values between two trees
PPKr	Recalibrated probability product kernel	computes the probability product kernel on preprocessed spectra

**Table 2:** Comparison of the top-k accuracy between CSI:FingerID, IOKR Unimkl and IOKRfusion in the negative and positive ionization modes. The highest top-k accuracies are shown in boldface.

Method	Negative Mode			Positive Mode		
	Top-1	Top-5	Top-10	Top-1	Top-5	Top-10
CSI:FingerID	31.9	60.2	69.9	36.0	67.5	76.5
IOKR Unimkl - Linear	30.1	58.8	68.6	34.9	66.9	76.0
IOKR Unimkl - Tanimoto	31.0	60.0	69.7	35.2	67.6	76.5
IOKR Unimkl - Gaussian	31.0	60.3	69.6	35.0	67.7	76.3
IOKR Unimkl - Gaussian Tanimoto	30.9	61.0	70.5	33.9	66.5	75.2
IOKRfusion - only IOKR scores	28.4	57.0	67.2	33.5	64.4	73.4
IOKRfusion - only IOKRreverse scores	30.1	60.4	71.4	37.6	69.2	77.9
IOKRfusion - all scores	**32.1**	**62.4**	**71.8**	**37.8**	**69.7**	**78.4**

**Table 3:** Running times for the training and the test steps.

Method	Training Time	Test Time
IOKR - linear	0.85 s	1 min 15 s
IOKR - tanimoto	3.9 s	7 min 40 s
IOKR - gaussian	7.2 s	8 min 38 s
IOKR - gaussian-tanimoto	7.6 s	8 min 44 s
IOKRreverse - linear	3.9 s	28 min 20 s
IOKRreverse - tanimoto	4.1 s	33 min 57 s
IOKRreverse - gaussian	7.4 s	34 min 49 s
IOKRreverse - gaussian-tanimoto	7.5 s	35 min 4 s
IOKR Unimkl - linear	4.3 s	1 min 10 s
IOKR Unimkl - tanimoto	8.7 s	7 min 52 s
IOKR Unimkl - gaussian	11.7 s	8 min 28 s
IOKR Unimkl - gaussian-tanimoto	11.9 s	8 min 42 s
IOKRfusion	3 min 3 s	0.1 s

## Supplementary Materials

The following are available online at https://www.mdpi.com/2218-1989/9/8/160/s1, Figure S1: Boxplots of the input kernel values for pairs of MS/MS spectra having the same molecular structure.
