# Supplementary material for: Improved Small Molecule Identification through Learning Combinations of Kernel Regression Models
Source: Metabolites. 2019 Aug 1;9(8):160. doi: 10.3390/metabo9080160 (PMC6724104; doi:10.3390/metabo9080160)
Supplement: Supplementary file 1 [file metabolites-09-00160-s001.pdf]

Article

Supplementary material: Improved small molecule identification through learning combinations of kernel regression models

Céline Brouard [1]*, Antoine Bassé[2], Florence d'Alché-Buc[2] and Juho Rousu [3,†]

[1] Unité de Mathématiques et Informatique Appliquées de Toulouse, UR 875, INRA, Castanet Tolosan, France; celine.brouard@inra.fr
[2] LTCI, Télécom Paris, Institut Polytechnique de Paris, France; florence.dalche@telecom-paris.fr
[3] Helsinki Institute for Information Technology HIIT, Department of Computer Science, Aalto University, Espoo, Finland; juho.rousu@aalto.fi
* Correspondence: celine.brouard@inra.fr
† Part of this work was commenced during J.R.'s research visit to Telecom ParisTech.

Version July 30, 2019 submitted to Metabolites

1. Difference in behavior between spectra with positive ionization mode and spectra in negative ionization mode

In Figure 1 we visualize the values of the different input kernels for pairs of spectra that have the same molecular structure. We visualize separately the kernel values for pairs of spectra both in positive ionization mode, both in negative ionization mode and with different ionization modes. We observe on the figure that for most of the kernels, spectra corresponding to the same structure but measured with different ionization modes are generally more dissimilar than when measured with the same ionization mode. This difference is particularly important in the case of the Root Loss Intensity (RLI) kernel. This is why we decided to consider the two types of spectra as two different datasets in the paper.

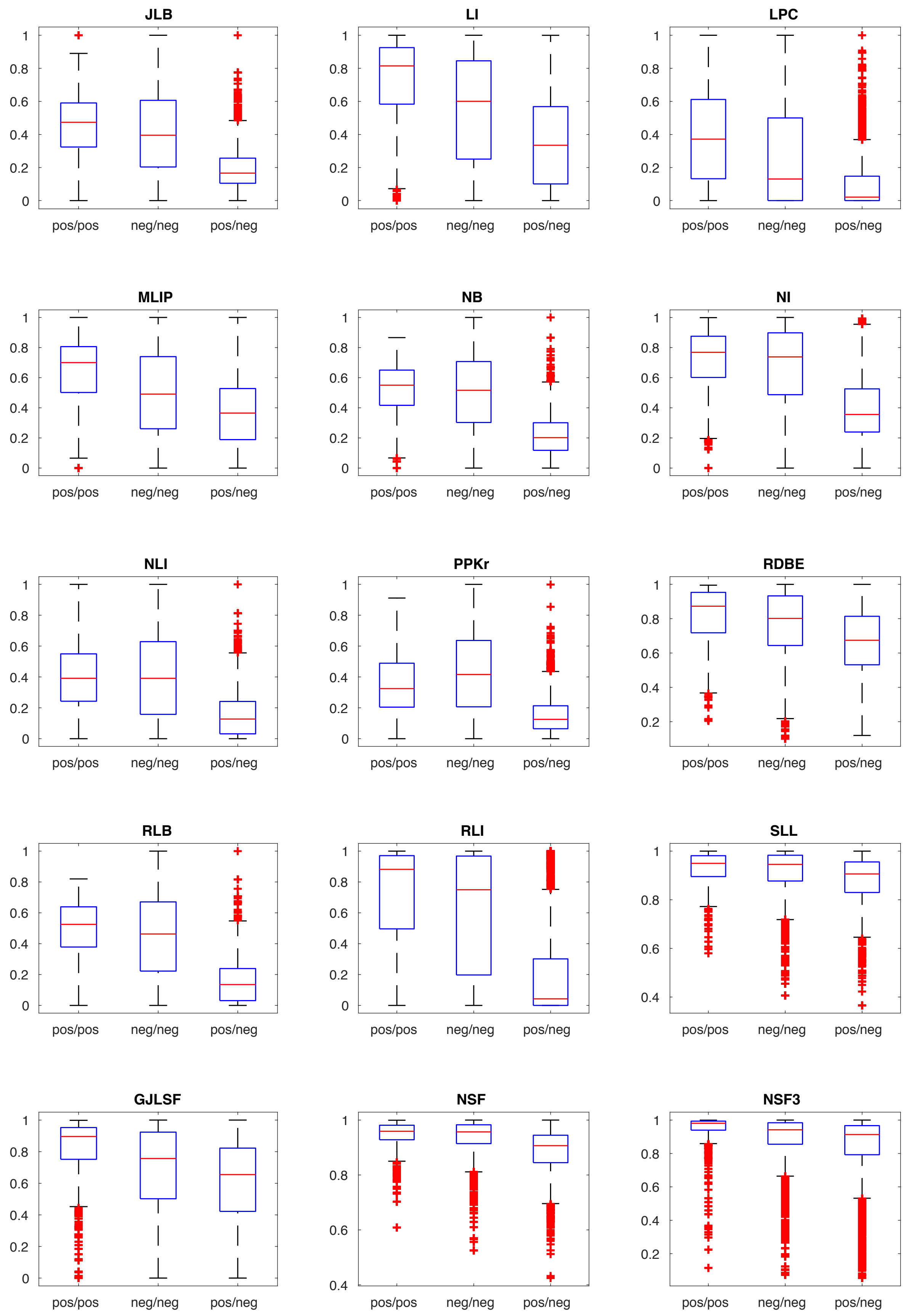

Figure 1. Boxplots of the input kernel values for pairs of MS/MS spectra having the same molecular structure. Each subplot corresponds to one input kernel and contains three boxplots. The first one corresponds to pairs of spectra where each spectrum has a positive ionization mode, the second one to pairs where each spectrum has a negative ionization mode and the third one to pairs of spectra with different ionization modes (one is positive and the other is negative).
